# Targetome profile of hsa-miR-93-5p is resistant to isoform formation in prostate adenocarcinoma

**DOI:** 10.7717/peerj.20642

**Published:** 2026-02-16

**Authors:** Anton Zhiyanov, Ivan Kirillov, Roman Suvorov, Diana Maltseva, Alexander Tonevitsky

**Affiliations:** 1 Faculty of Biology and Biotechnology, Higher School of Economics, Moscow, Russia; 2 Shemyakin-Ovchinnikov Institute of Bioorganic Chemistry, Moscow, Russia

**Keywords:** miR-93-5p, 5’-isomiR targets, Seed region, Dual targeting

## Abstract

MicroRNAs (miRNAs) and their isoforms, known as isomiRs, are important regulators of tumorigenesis that act as post-transcriptional modulators of gene expression. Among these, 5’-isomiRs—generated through imprecise cleavage during miRNA biogenesis—exhibit altered seed regions compared to their canonical counterparts, potentially leading to distinct targetomes. Consequently, 5’-isomiRs may exert biological functions that differ substantially from those of the corresponding canonical miRNAs. Despite growing recognition of their potential significance, the functional roles of 5’-isomiRs remain largely uncharacterized for most miRNAs. In this study, we investigated the targetome divergence between canonical miRNAs and their 5’-isomiRs, focusing on hsa-miR-93-5p, a miRNA with a well-established oncogenic role in prostate adenocarcinoma. Target transcripts of the 5’-isomiRs were identified using a shRNA-based overexpression system. Bioinformatic analysis revealed a substantial overlap between the targets of the 5’-isomiRs and the canonical miRNA. This overlap was attributed to the co-occurrence of both canonical and shifted seed motifs within the same mRNA targets. Notably, hsa-miR-93-5p ranked among the top miRNAs with a relatively high number of targets transcripts containing both seed motifs, suggesting a unique dual-targeting capacity.

## Introduction

Prostate cancer (PCa) is the second most commonly diagnosed malignancy and the fifth leading cause of cancer-related mortality among middle-aged and older men worldwide (Bray et al., 2024). Recent studies have highlighted that non-coding RNA (ncRNA) networks exert multi-layered control over PCa progression, integrating transcriptional, post-transcriptional and translational regulation. In particular, enhancer RNAs (eRNAs) have emerged as active regulatory molecules rather than passive by-products of enhancer activity. eRNA-driven programs were shown to modulate ferroptosis sensitivity in PCa cells by linking enhancer-dependent chromatin activation to androgen-dependent regulatory programs and cell-death pathways, thereby establishing a direct connection between epigenomic remodeling and cancer cell vulnerability (Ma et al., 2025).

In parallel, growing evidence demonstrates that PIWI-interacting RNAs (piRNAs) and PIWI proteins participate in oncogenic translation control beyond their canonical role in genome stability. piRNA-mediated repression of target mRNAs is frequently coupled to RNA modifications such as m6A or altered RNA circularization and can fine-tune protein synthesis of key regulators of proliferation, invasion, and therapy resistance in prostate tumors (Ben et al., 2024; Jia et al., 2022).

MicroRNAs (miRNAs) and their isoforms represent an additional layer of mRNA expression regulation at the post-transcriptional level in PCa (Yuan et al., 2024; Wagner, Meese & Keller, 2024). MiRNAs are a class of small non-coding RNAs that regulate mRNAs *via* complementary base pair binding (Hobert, 2008; Siomi & Siomi, 2010). A binding of a mature miRNA, in complex with an Argonaute protein, directly to a target mRNA, leads to the mRNA degradation or translational repression (Nilsen, 2007).

During miRNA biogenesis, two RNase III family nucleases—Drosha and Dicer—sequentially cleave the miRNA hairpin structure to generate a precursor miRNA (pre-miRNA) and, ultimately, a single-stranded mature miRNA (Kim, Kim & Kim, 2016). Inaccurate cleavage by these enzymes can result in the addition or removal of one or more nucleotides at the 5′-end of the mature miRNA. Such variations alter the seed region—nucleotides 2 through 7 or 8 from the 5′-end—which is critical for target recognition (Gebert & MacRae, 2018; Neilsen, Goodall & Bracken, 2012). These alternatively processed miRNAs with shifted seed regions are referred to as 5′-end miRNA isoforms, or 5′-isomiRs.

Base pairing within the seed region is the primary determinant of miRNA-mRNA target interaction (Bartel, 2009), and therefore therefore forms the basis of most bioinformatic target prediction models (Chen & Wang, 2019; Agarwal et al., 2015). As a result, these models typically predict limited overlap between the targetomes of canonical miRNAs and their 5′-isomiRs (Nersisyan et al., 2022a; Nersisyan et al., 2022b). This prediction has been supported experimentally (Salem et al., 2016; Li et al., 2022). For instance, a recent study demonstrated that 5′-isomiRs of hsa-miR-183-5p functioned as tumor suppressors in triple-negative breast cancer (TNBC) cell lines (Li et al., 2022). Notably, the hsa-miR-183-5p|+2 isoform (shifted by two nucleotides at the 5′-end) exhibited stronger antiproliferative effects than the canonical form, reducing cell proliferation and inhibiting cell cycle progression in TNBC models.

In this study, we focus on hsa-miR-93-5p, a miRNA with a well-established oncogenic role in various cancer types (Yang et al., 2018; Pan et al., 2021). Recent research has shown that its regulatory activity is notably elevated in PCa relative to other miRNAs (Zhiyanov et al., 2023). In our previous work (Maltseva et al., 2024), we constructed several short hairpin RNAs (shRNAs) encoding hsa-miR-93-5p and achieved stable overexpression of its 5′-isomiRs in PC-3 prostate adenocarcinoma cell line. Subsequent mRNA-seq analysis, we identified 54 mRNAs that were significantly downregulated following this overexpression.

In the present study, we extend this analysis by examining public mRNA-seq data from PCa tissues (TCGA-PRAD) to explore the relationship between these identified mRNAs and the canonical form of hsa-miR-93-5p.

## Materials and Methods

### Cell culturing and lentiviral transduction

The human embryonic kidney cell line HEK293T was grown in DMEM medium containing 4.5 g/L glucose (PanEco, Moscow, Russia) supplemented with 4 mM L-glutamine (PanEco, Moscow, Russia), 10% fetal bovine serum (Gibco, Waltham, MA, USA), 1% penicillin and streptomycin (Gibco, Waltham, MA, USA) and cultured under the standard cell culture conditions (37 °C, in a humidified atmosphere with 5% CO_2_). Stable overexpression of has-miR-93-5p isoforms was achieved by an shRNA-mediated method. For this, double-stranded DNA fragment (Fr-5′-caccCTACCTGCACGAACAGCACTTTGTTCAAGAGACAAAGTGCTGTTCGTGCAGGTAGttttt, Rev-5′-ctctaaaaaCTACCTGCACGAACAGCACTTTGTCTCTTGAACAAAGTGCTGTTCGTGCAGGTAG) containing hsa- miR-93-5p sequence was cloned into the BsmBI restriction sites of the LentiGuide-Hygro lentiviral vector (was a gift from Caroline Goujon, Addgene #139462, UK) with deleted sgRNA scaffold. The same vector containing a short sequence of sgRNA (5′-TTCTCTTGCTGAAAGCTCGA) was used to generate a transduced control cell line (Ctrl). The shRNA transcription starts were located at precisely 23 nucleotides away from the TATA box in all vectors used in the experiment. Nucleotide sequences of the obtained lentiviral vectors were confirmed by Sanger sequencing. Lentiviral particles were obtained as cell-free supernatants by transient transfection of HEK-293T as described in Maltseva et al. (2020) and Everest-Dass et al. (2023) using Lipofectamine 3000 (Invitrogen, Carlsbad, CA, USA). Lentiviral vectors based on pLentiGuide-Hygro were packaged using the second-generation packaging plasmid pCMV-dR8.2 dvpr (Addgene #8455, UK) and pCMV-VSV-G (Addgene #8454, UK) expressing the envelope protein of vesicular stomatitis virus (Stewart et al., 2003). The supernatant was harvested 24 h and 48 h after transfection, 0.45 µm filtered and was immediately used for transduction or stored at −80 °C. HEK293T cells were plated at 8 × 105 cells in two mL medium in each well of a 6-well plate 24 h before transduction. After viral particles containing supernatant were added to the cells, the medium was replaced in 3 days, cells were seeded into a 25 cm^2^ flask, and hygromycin was added at a concentration of 150 µg/mL. The hygromycin selection was carried out for two weeks. Thus, two lines were obtained: HEK293T cells stably expressing hsa-miR-93-5p isoforms (HEK293T-shMiR-93) and HEK293T control line transduced by lentiviral particles with control vector (HEK293T-Ctrl). The overexpression of hsa-miR-93-5p 5′-isomiRs in HEK293T-shMiR-93 cells was confirmed by qPCR.

### MiRNA expression analysis by qPCR

The analysis was performed as previously described in Maltseva et al. (2024). Specifically, according to the manufacturer’s instructions, the total RNA was isolated from about 1 × 106 cells using miRNeasy Mini Kit (Qiagen, Hilden, Germany). All RNA samples were treated with DNase I during the isolation procedure. The RNA yield was determined by UV absorbance using a NanoDrop 1000 spectrophotometer (Peqlab, Erlangen, Germany). The RNA quality was assessed by analyzing the ribosomal RNA integrity number (RIN) on an Agilent 2000 Bioanalyzer with the RNA 6000 Nano kit (Agilent Technologies, Santa Clara, CA, USA). Based on the fact that RNA Polymerase III inserts additional uracils at a 3′-end of shRNA during transcription termination (Gao, Herrera-Carrillo & Berkhout, 2018), to assess hsa-miR-95-5p isoform overexpression, primers for its 3′-canonical form (hsa-miR-95-5p|0|0) as well as for its isoforms containing two (hsa-miR-95-5p|0|+2U) and three uracils (hsa-miR-95-5p|0|+3U) at the 3′-end were used. MiRNAs hsa-miR-191-5p and hsa-miR-182-5p were used as reference ones. Primer sequences generated using the miRprimer2 software (Busk, 2014) are listed in Table S5. Reverse transcription of total RNA was performed according to Busk (2014). In brief, 750 ng of total RNA were poly(A) tailed by poly(A) polymerase (New England Biolabs, Ipswich, MA, USA) and reverse transcribed by SuperScrip VILO cDNA Synthesis Kit (Invitrogen, Carlsbad, CA, USA) in a single tube reaction using RT-primer (5′-CAGGTCCAGTTTTTTTTTTTTTTTVN, V = A, C, and G; N = A, C, G, and T). The cDNA was 5 times diluted before the qPCR reaction. Quantitative PCR analysis was carried out using the SYBR Green 5x qPCRmix-HS SYBR reaction mix (Evrogen, Russia): 2.5 µl of diluted cDNA, 1.2 µl of 5 µM specific primer mix, and 5 µl of SYBR Green qPCRmix were mixed in a final volume of 25 µl. All RNA samples were analyzed in triplicate and averaged. MiRNA isoforms’ expressions were normalized to the reference miRNAs, and data were processed based on the ΔΔCt method (Livak & Schmittgen, 2001).

### Reporter plasmid construction

The p.UTA.3.0 empty vector (Addgene #82447), which contains two independent fluorescent proteins—GFP and TurboRFP—each expressed from separate promoters (Lemus-Diaz et al., 2017), was used to generate dual fluorescence reporter systems. To construct a reporter for detecting the specific activity of hsa-miR-93-5p|+3/+4 5′-isomiRs (referred to as reporter #Iso), a 87 bp double-stranded DNA fragment containing three tandem target sites for the isoforms was inserted into the 3′-UTR of the RFP gene using site-directed mutagenesis. Briefly, two overlapping DNA oligonucleotides (sequences provided in Table S6) were used as primers for PCR amplification. The primer annealing scheme and full sequence of the resulting 3′-UTR fragment are shown in Fig. S3. PCR amplification was carried out using Q5 High-Fidelity DNA Polymerase (New England Biolabs, Ipswich, MA, USA). The target sites within the inserted fragment were fully complementary to hsa-miR-93-5p|+3/+4 5′-isomiRs, except for the CU nucleotides at positions 8–9 of each site, which were substituted with GA to prevent off-target interactions with the seed regions of other miRNAs endogenously expressed in HEK293T cells (see Fig. S3). As a control, a second reporter (reporter #Iso-mut) was created by introducing mutations into the seed regions of the isoform binding sites, thereby abolishing RFP repression mediated by hsa-miR-93-5p|+3/+4 5′-isomiRs. The correct orientation and sequence of the inserted isoform binding sites were confirmed by Sanger sequencing.

### Reporter assay

HEK293T-shMiR-93 and HEK293T-Ctrl cells were seeded in 24-well plates at a density of 60,000 cells per well. Six hours later, the cells were transfected with reporter plasmids using Lipofectamine 3000 (Invitrogen, Carlsbad, CA, USA). The transfection complexes were prepared in Opti-MEM I Reduced Serum Medium (Invitrogen, Carlsbad, CA, USA). Fluorescence images were captured at 24 h and 46 h post-transfection using a Zeiss Axio Observer fluorescence microscope equipped with an HXP 120 C illuminator, utilizing the AlexaFluor488 and TurboRFP channels. At 48 h post-transfection, fluorescence intensity was quantified using a SinoCyte X flow cytometer (Biosino). The functional activity of the isoforms was evaluated by comparing the normalized GFP/RFP fluorescence intensity ratios between HEK293T-shMiR-93 and HEK293T-Ctrl cells transfected with the #Iso and #Iso-mut reporters. Functional isoform activity is expected to reduce RFP expression, resulting in an increased GFP/RFP fluorescence ratio in HEK293T-shMiR-93 cells transfected with the #Iso reporter compared with HEK293T-Ctrl cells. In contrast, for the #Iso-mut reporter, the GFP/RFP ratios in both cell lines are expected to remain similar. For each cell line, normalization of the GFP/RFP fluorescence intensity ratio for the #Iso and #Iso-mut reporters was performed by dividing the obtained ratio by the GFP/RFP ratio of the parental p.UTA.3.0 plasmid, which lacks binding sites for hsa-miR-93-5p|+3/+4 5′-isomiRs, within the same cell line.

### Analysis of prostate adenocarcinoma tissues RNA-seq data

MiRNA-Seq and mRNA-Seq read count tables for PCa tissues (TCGA-PRAD) were downloaded from Xena Browser. The data were processed as follows.

First, mRNA-Seq samples were normalized for the size factors and transformed to the median-of-ratios scale according to the standard DESeq2 pipeline (Love, Huber & Anders, 2014). Gene length normalization was then applied to calculate fragments per kilobase of transcript per million mapped reads (FPKM), followed by log-transformation: log_2_(1 + FPKM).

To remove lowly expressed genes, the following filtering procedure was used: genes were ranked by median expression in descending order, and a cumulative sum of median expression was computed. Genes contributing less than 99% of the cumulative expression were excluded from further analysis.

MiRNA-Seq data were processed similarly, except that gene length normalization was omitted. Expression values were log-transformed as log_2_(1 + RPM), where RPM refers to reads per million mapped reads.

Spearman’s rank-order correlation coefficients and associated *p*-values (testing the null hypothesis of zero correlation) were calculated using expression data from 490 primary tumor samples in TCGA-PRAD to assess the association between mRNA targets and miRNA expression. *P*-values were adjusted using the Benjamini–Hochberg method to control the false discovery rate.

### MiRNA isoforms notation

We adopted the standard isomiR notation proposed in Loher et al. (2021). A numeric suffix following the “|” symbol denotes the number of nucleotide shifts from the canonical 5′-end in the 5′ to 3′ direction (*i.e.,* 5′-isomiR notation).

Seed region complementarity within the mRNA sequence is known to be a key determinant of miRNA-mediated regulation (Bartel, 2009). However, the regulatory effectiveness depends on the seed length. In accordance with widely used classification schemes (Bartel, 2009; Agarwal et al., 2015), we considered two types of seed matches: “6mer” and “7mer-m8”, corresponding to nucleotides 2–7 and 2–8 from the 5′-end of the mature isomiR, respectively. The GRCh38 reference genome and GENCODE release 34 annotation (Mudge et al., 2024) were used to extract mRNA nucleotide sequences and identify occurrences of seed matches.

### Identification of hsa-miR-93-5p 5′-isomiRs targets

MiRNA-seq and mRNA-seq data from hsa-miR-93-5p-transduced PC-3 cell lines were retrieved from the NCBI Gene Expression Omnibus (GEO) under accession number GSE241303. Detailed descriptions of the shRNA constructs used to express hsa-miR-93-5p are provided in our previous work (Maltseva et al., 2024).

Based on that study, 54 mRNAs were significantly downregulated following overexpression of the hsa-miR-93-5p|+3/+4 5′-isomiRs. A complete list of these mRNAs, including expression levels and log_2_(fold change) values, is available in Table S1.

### Statistical analysis

Spearman’s rank-order correlation was used to identify negatively correlated (putatively repressed) targets of canonical hsa-miR-93-5p. *P*-values from zero-correlation tests were adjusted using the Benjamini–Hochberg procedure. Enrichment of seed regions within experimentally identified mRNAs was assessed using odds ratios and Fisher’s exact test. The Mann–Whitney U test was employed to compare the distribution of correlation coefficients between mRNAs with and without “6mer” seed matches. Overlap between canonical and 5′-isomiR target sets was quantified using the Jaccard index, defined as the size of their intersection divided by the size of their union. Statistical significance was set at *p*-value <0.05.

## Result

### The 5′-isomiRs hsa-miR-93-5p|+3/+4 are functionally active in cells

**Figure 1:**
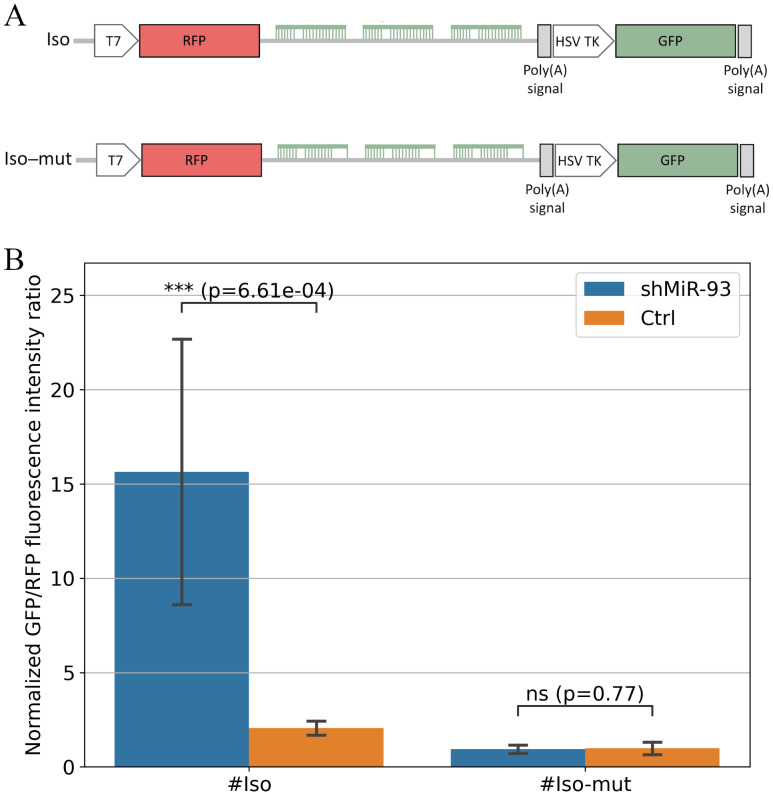
Functional activity of hsa-miR-93-5p|+3/+4 5′-isomiRs. (A) Schematic representation of the dual fluorescence reporters used in this study, containing either a perfectly matched or mutated seed region for hsa-miR-93-5p|+3/+4 5′-isomiRs. (B) Normalized GFP/RFP fluorescence intensity ratios. Suppression of RFP expression leads to an increased GFP/RFP ratio, as observed in the HEK293T-shMiR-93 cells (shMiR-93) transfected with the #Iso reporter compared with the control HEK293T-Ctrl cells (Ctrl). In contrast, for the #Iso-mut reporter, GFP/RFP ratios did not differ between HEK293T-shMiR-93 and HEK293T-Ctrl. This indicates that RFP repression by the #Iso reporter is specifically mediated by 5′-isomiRs to their target sites within the 3′-UTR of the RFP mRNA. For each cell line, GFP/RFP ratio for the #Iso and #Iso-mut reporters were normalized to the ratio obtained with the parental plasmid p.UTA.3.0, which lacks binding sites for hsa-miR-93-5p|+3/+4 5′-isomiRs. *** symbols mean *p*-value ≤1∗10^−3^.

One objective of the present study was to confirm the functional activity of of hsa-miR-93-5p 5′-isomiRs generated upon shRNA-mediated overexpression using an RNA Polymerase III (Pol III)-based expression cassette. In our previous study (Maltseva et al., 2024), Ago2 immunoprecipitation showed that the overexpressed hsa-miR-93-5p|+3/+4 5′-isomiRs are incorporated into the Ago2-containing RISC complex, indicating their potential functional competence in regulating mRNA targets. These 5′-isomiRs differ from the canonical miRNA by specific nucleotide variations –specifically, the presence of A or G at position 1 instead of the canonical C, which is more favored by Argonaute proteins (Duchaine & Fabian, 2018), and the presence of additional uracils at the 3′-end added by RNA Polymerase III (Maltseva et al., 2024). To assess whether these nucleotide variations affect 5′-isomiR activity, we performed a dual-fluorescence reporter assay. A reporter plasmid containing three binding sites for the hsa-miR-93-5p|+3/+4 5′-isomiRs (reporter #Iso; Fig. 1A) was constructed. This design was expected to induce strong repression of RFP fluorescence upon the 5′-isomiR overexpression (Brustikova et al., 2018), while GFP fluorescence would remain unchanged. As a control, a mutant reporter lacking functional 5′-isomiR binding sites (reporter #Iso-mut; Fig. 1A) was generated, which was expected to abolish RFP repression by the 5′-isomiRs.

In our previous work (Maltseva et al., 2024), we observed the formation of hsa-miR-93-5p 5′-isomiRs in two PCa cell lines, PC-3 and DU-145, suggesting that 5′-isomiR generation is independent of cell line type and likely governed by intrinsic features of shRNA or pre-miRNA processing. This conclusion is further supported by unpublished miRNA sequencing data from hepatocellular carcinoma HepG2 cells, transduced with the same lentiviral particles encoding hsa-miR-93-5p used in the prostate cancer cell lines, which also produced similar isomiRs (D Maltseva, 2025, unpublished data). Based on these findings, we overexpressed hsa-miR-93-5p 5′-isomiRs in HEK293T cells—chosen for their high transfection efficiency—using the same lentiviral particles. The resulting cell lines HEK293T-shMiR-93 (overexpressing hsa-miR-93-5p|+3/+4 5′-isomiRs) and HEK293T-Ctrl (control cell line) were transfected with reporter plasmids #Iso and #Iso-mut. As expected, in HEK293T-shMiR-93 cells, the #Iso reporter exhibited strong repression relative to reporter #Iso-mut (Fig. 1B), while neither reporter showed repression in the HEK293T-Ctrl cells. These results confirm the functional activity of hsa-miR-93-5p|+3/+4 5′-isomiRs.

### The experimentally identified targets of 5′-isomiRs hsa-miR-93-5p| +3/+4 are anticorrelated with the canonicial isoform

As previously described (Maltseva et al., 2024), PC-3 (Prostate Adenocarcinoma) cell lines were transduced with shRNAs encoding hsa-miR-93-5p, resulting in a strong overexpression of its 5′-isomiRs hsa-miR-93-5p|+3 and hsa-miR-93-5p|+4. These isomiRs showed a >1,000-fold increase in mean expression (log_2_(fold change) > 10), reaching 1.5% and 2.3% of total miRNA expression, respectively.

Consequently, 54 highly expressed mRNAs were significantly and consistently downregulated across all replicates (log_2_(fold change) < 0, DESeq2-adjusted *p*-value <0.05).

Most of these downregulated mRNAs contained a “7mer-m8” seed region corresponding to the +3/+4 5′-isomiRs but lacked the “7mer-m8” seed of the canonical miRNA. This finding was supported by enrichment analysis, which showed a statistically significant overrepresentation of the 5′-isomiR seed regions in both 3′-UTRs and full mRNA sequences (Fisher’s exact test, adjusted *p*-value <0.05; Table S1).

Further inspection of TCGA-PRAD data revealed that 47 out of the 54 mRNAs were expressed at significant levels in primary tumor samples, while ohter 7 (*PCSK9*, *AHNAK2*, *MUC5A*, *FGFBP1*, *PTPRH*, *PEAR1*, *KRT75*) had log_2_(1 + FPKM) < 1. Among the expressed mRNAs, the majority were significantly anticorrelated with canonical hsa-miR-93-5p expression (Spearman’s correlation <0, adjusted *p*-value <0.05). Fisher’s exact test across all mRNAs expressed in primary tumors further showed that these 47 mRNAs were significantly overrepresented among anticorrelated mRNAs (odds ratio =4.24, Fisher’s exact test *p*-value <0.05).

These findings challenge the conventional assumption that canonical miRNAs and their 5′-isomiRs have distinct targetomes, suggesting a potential overlap in regulatory targets. Notably, in addition to the fact that hsa-miR-93-5p|+3 isomiR was significantly downregulated in PCa tissues. Both +3/+4 5′-isomiRs were expressed at low levels in the tumor samples accounting for up to 2% of the canonical isoform’s expression (Fig. S1), and did not correlate with the canonical form (Spearman’s correlation =0.1 for both isomiRs).

### The presence of “6mer” seed region is sufficient for hsa-miR-93-5p-mediated regulation

**Figure 2:**
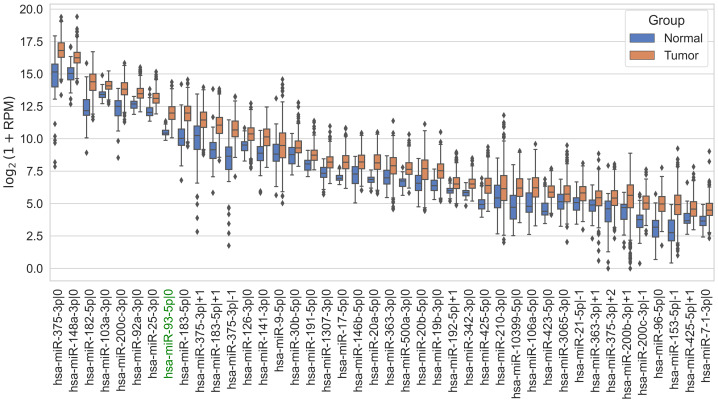
The most expressed up-regulated 5’-isomiRs in the normal prostate and adenocarcinoma tissues.

**Figure 3:**
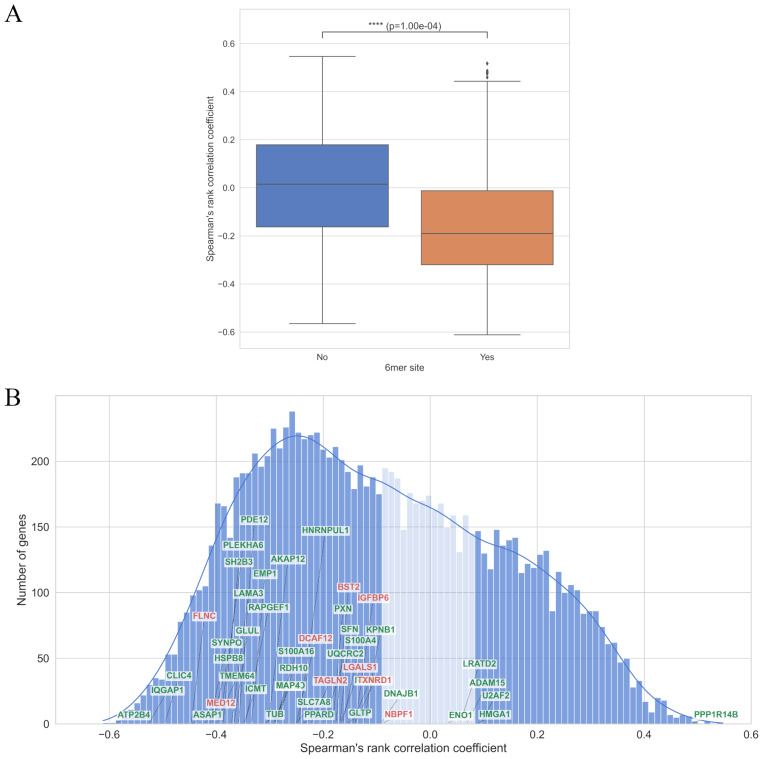
(A) The presence of “6mer” seed region in mRNA is associated with stronger negative correlation of mRNA with hsa-miR-93-5p in the prostate adenocarcinoma tissues. (B) The majority of the experimentally validated hsa-miR-93-5p|+3/+4 targets (38 mRNAs marked in green) also contain “6mer” seed region of the canonical form. **** symbols mean *p*-value ≤1∗10^−4^.

Many target prediction tools consider the “6mer” seed region sufficient for miRNA-mediated regulation. Analysis of TCGA-PRAD data supports this notion: hsa-miR-93-5p was among the most highly expressed and upregulated miRNAs in PCa tissues (Fig. 2). Correspondingly, mRNAs containing the canonical “6mer” seed site exhibited significantly stronger anticorrelation with hsa-miR-93-5p expression compared to genes without this seed (Mann–Whitney U test, *p*-value <0.05; Fig. 3A).

Among the 47 identified downregulated mRNAs, 38 contained a canonical “6mer” seed match (Fig. 3B). Enrichment analysis confirmed a statistically significant overrepresentation of canonical “6mer” sites in this set (odds ratio =3.51, Fisher’s exact test *p*-value <0.05).

### Targets of hsa-miR-93-5p 5′-isomiRs exhibit one of the highest intersections

Considering previous results, we investigated whether overlap between canonical and 5′-isomiR targets *via* shared “6mer” seed regions is a general feature of highly expressed miRNAs.

We first selected 64 most abundant miRNAs (Fig. S2), which together accounted for 99% of total miRNA expression in PCa tissues (TCGA-PRAD dataset). For each miRNA, potential 5′-isomiR targets were identified based on the presence of “7mer-m8” sites, while canonical targets were identified based on “6mer” seed matches.

**Figure 4:**
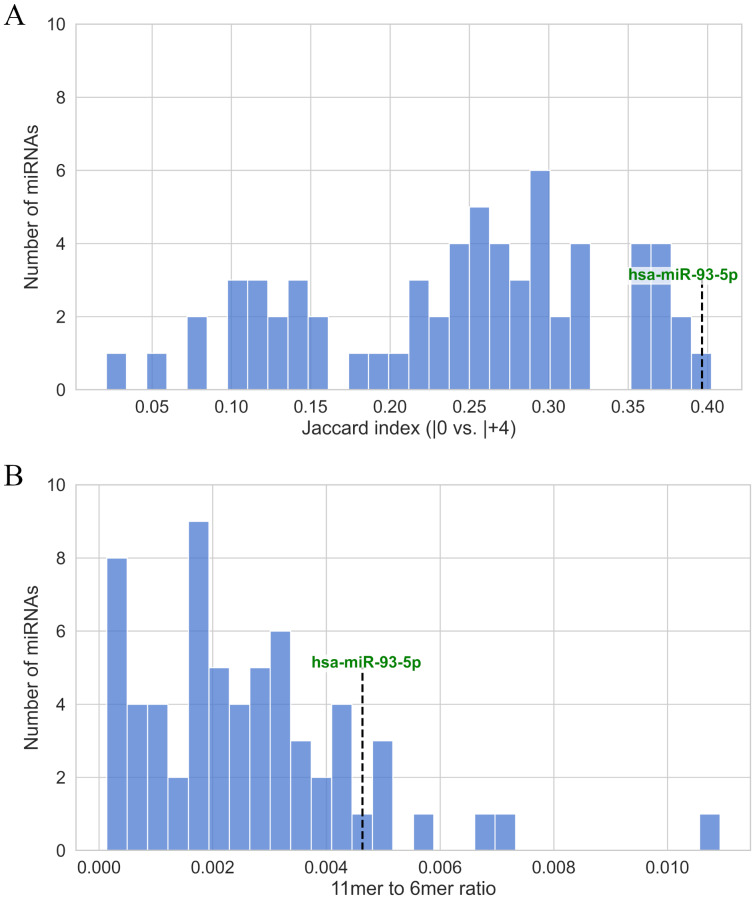
(A) Jaccard index of the canonical and 5′-isomiR target sets. (B) The proportion of canonical “6mer” seed sites that could be extended to match the “7mer-m8” seed of the hsa-miR-93-5p|+4 isomiR.

Jaccard indices, defined as the ratio of the intersection to the union of the canonical and 5′-isomiR target sets, were calculated for each miRNA. As shown in Fig. 4A, hsa-miR-93-5p and its +4 5′-isomiR exhibited the highest Jaccard index among all analyzed miRNAs. It belonged to a cluster of 11 miRNAs with Jaccard indices ranging from 0.35 to 0.40 (Table S2). The +3 5′-isomiR also demonstrated considerable overlap with canonical targets, with its Jaccard index fell in the 73rd percentile relative to all other miRNAs (Table S3).

We further assessed the proportion of canonical “6mer” seed sites that could be extended to match the “7mer-m8” seed of the hsa-miR-93-5p|+4 isomiR (*i.e.,* 11-nucleotide matches in total). Although this proportion was generally low across all miRNAs (Fig. 4B), hsa-miR-93-5p ranked in the top 15%. The following miRNAs showed even higher extension potential: hsa-let-7e−5p, hsa-miR-26b-5p, hsa-miR-26a-5p, hsa-miR-145-5p, hsa-miR-203a-3p, hsa-miR-125a-5p, hsa-miR-125b-5p and hsa-miR-141-3p (see Table S4).

## Discussion

In our previous experiment (Maltseva et al., 2024), we identified 54 mRNA targets that were significantly downregulated following the overexpression of hsa-miR-93-5p 5′-isomiRs. At the same time, analysis of publicly available TCGA-PRAD data revealed that a substantial portion of these mRNAs also showed negative correlation with the expression of the canonical hsa-miR-93-5p. This observation challenges the conventional assumption that 5′-isomiRs and their canonical miRNA counterparts regulate largely distinct sets of genes.

Emerging evidence now demonstrate that 5′-isomiRs are not merely biochemical by-products of imprecise processing but can drive phenotypes distinct from those mediated by canonical miRNAs. A key underlying mechanism is seed shifting, whereby the gain or loss of even a single nucleotide at the 5′-end redefines the 2–7/8 nt seed region, thereby redirecting target specificity (Bartel, 2009). The functional relevance of this phenomenon was first demonstrated by Tan et al. (2014), who showed that 5′-isomiRs of several conserved miRNAs selectively repress alternative mRNA sets and, in some cases, display stronger evolutionary conservation than the corresponding canonical miRNAs. More recently, the authors of Li et al. (2022) provided direct experimental evidence that the +2 5′-isomiR of hsa-miR-183-5p acts as a tumor suppressor in TNBC by targeting the transcription factor E2F1, whereas the canonical form shows only weak activity. In contrast, both the canonical and the +1 5′-isomiR forms of hsa-miR-140-3p act synergistically in breast tumor suppression by inhibiting distinct tumor-associated processes (Salem et al., 2016; Bhardwaj et al., 2018). Furthermore, (Park et al., 2022) demonstrated that the RNA-binding protein hnRNPC remodels hsa-miR-21 biogenesis in liver cancer cells, producing abundant +1 and -1 5′-isomiRs that activate a distinct oncogenic program. Interestingly, +3, +4, +5, and +6 5′-isomiRs of hsa-miR-21-5p have been associated with protective effects in lung cancer, whereas canonical hsa-miR-21-5p is a well-known promoter of metastasis (Hsieh et al., 2021). Collectively, these findings establish that alternative seed specification of 5′-isomiRs can yield non-redundant and functionally significant biological outcomes in tumorigenesis.

It is also important to note that isomiRs are differentially expressed in a wide range of biological contexts beyond cancer. For example, isomiRs of hsa-miR-221 and hsa-miR-30a are expressed at different levels in male and female samples (Guo et al., 2016; Loher, Londin & Rigoutsos, 2014). IsomiRs additionally exhibit population-dependent (Loher, Londin & Rigoutsos, 2014) and tissue-specific expression patterns (Aparicio-Puerta et al., 2022; Keller et al., 2021). Moreover, 5′-shifted isomiRs have been implicated in gene expression regulation in type 2 diabetes (Baran-Gale et al., 2013) and cardiovascular disease (Van der Kwast et al., 2020), and their involvement in neurodegenerative disorders is also under discussion (Wagner, Meese & Keller, 2024).

Within this landscape, hsa-miR-93-5p represents a mechanistically distinct case. Since the +3 and +4 5′-isomiRs of hsa-miR-93-5p possess shifted seed sequences compared to the canonical form, their binding is expected to occur at distinct locations within mRNAs. However, our analysis revealed that for many of the 54 downregulated mRNAs, canonical seed matches—especially “6mer” motifs—were also present. These additional sites may explain the observed anticorrelation with the canonical isoform in tumor samples, despite the original experimental design specifically targeting 5′-isomiRs. This “dual-targeting architecture” places hsa-miR-93 in a minority class of miRNAs in which seed shifting does not rewire regulatory output, but instead reinforces repression of a shared mRNA set through parallel seed logics. The functional consequences of this architecture, *e.g.*, stabilized repression, buffering against processing noise, or more robust target silencing, warrant further investigation. It should also be noted that, in addition to the dual-seed enrichment mechanism proposed above, alternative explanations, such as partial complementarity-mediated off-target repression, cannot be fully excluded.

Interestingly, these results may reflect a broader targeting mechanism that extends beyond seed pairing alone. In this context, Shin et al. (2010) introduced the concept of “centered sites”, a class of functional miRNA target sites that do not require seed region pairing, but instead exhibit contiguous base pairing to the central region of the miRNA. Although such sites typically do not induce mRNA cleavage *in vivo*, they can effectively repress protein translation effectively. Given that the canonical and isomiR forms of hsa-miR-93-5p share high similarity in their central regions, it is plausible that centered pairing contributes to the overlapping regulatory effects observed in our study. This hypothesis could also explain why some mRNAs may appear as shared targets despite divergent seed sequences.

Moreover, we previously demonstrated that the regulatory relationship between miRNAs and their mRNA targets is broadly disrupted in PCa (Zhiyanov et al., 2023). For most highly expressed miRNAs, the typical inverse correlation with target mRNA expression is either weakened or completely lost in tumor samples. Remarkably, hsa-miR-93-5p stands out as a consistent exception to this trend—it retains significant anticorrelation with many of its predicted targets, indicating preserved regulatory activity. This robustness, coupled with its high expression levels, supports its biological relevance in PCa. Furthermore, hsa-miR-93-5p holds importance: its plasma concentration in blood plasma increases with disease progression, highlighting its potential as a non-invasive biomarker for prognosis and patient monitoring.

Taken together, our findings indicate that hsa-miR-93-5p represents a mechanistically distinct subclass of miRNAs in which 5′-end heterogeneity does not lead to divergent targetomes but rather amplifies repression of shared transcripts through seed-redundant targeting. These results highlight the potential clinical relevance of this miRNA and emphasize the need for further investigation of its functional role in PCa. This notion is supported by recent studies (Gómez-Acebo et al., 2025; Gazzaz et al., 2023). Current evidence suggests that hsa-miR-93-5p promotes PCa progression by enhancing cell proliferation, invasion, migration, and metastasis (Yang et al., 2019; Liu, Zhang & Wu, 2018).

A limitation of the present study is that our analysis focused on a single miRNA, hsa-miR-93-5p, and one isomiR class, and the functional relevance of the observed targetome stability was not experimentally validated in additional prostate-relevant models. It remains to be determined whether similar isomiR targetome stability can be observed in other miRNAs across other cancer types. Future studies will be required to determine whether the unusually low divergence observed for hsa-miR-93-5p represents an isolated case or reflects a more general regulatory principle.

## Supplemental Information

10.7717/peerj.20642/supp-1 Supplemental Information 1 MIQE checklist

10.7717/peerj.20642/supp-2 Supplemental Information 2 hsa-miR-93-5p 5′-isomiRs expression profile in prostate adenocarcinoma Differential expression analysis was performed using DESeq2.

10.7717/peerj.20642/supp-3 Supplemental Information 3 Highly expressed miRNAs in prostate adenocarcinoma

10.7717/peerj.20642/supp-4 Supplemental Information 4 Reporter constructs and primer annealing patterns The 3′-UTR sequences of the reporter constructs used in this study are shown. Also depicted are the annealing patterns of the primers used for site-directed mutagenesis to generate these constructs. Regions that are identical to the +3/+4 5′-isomiRs of hsa-miR-93-5p are highlighted in green and bold. Nucleotides that were intentionally altered to complementary bases –aimed at preventing binding by other miRNAs endogenously and constitutively expressed in HEK293T cells –are shown as underlined text. Spacer sequences separating the binding sites of the +3/+4 5′-isomiRs are highlighted in yellow, blue, and purple. Primer regions complementary to the p.UTA.3.0 plasmid backbone used to construct the reporters are highlighted in black. Mutations introduced into the seed regions to disrupt isomiR binding to the 3′-UTR of the RFP mRNA are highlighted in red.

10.7717/peerj.20642/supp-5 Supplemental Information 5 Targetome profile of hsa-miR-93-5p is resistant to isoform formation in prostate adenocarcinoma

10.7717/peerj.20642/supp-6 Supplemental Information 6 5′-isomiRs target identification analysis TCGA-PRAD –expression in *log*_2_(1 + *FPKM*)-scale in prostate adenocarcinoma primary tumor samples; PC-3_Control –expression in *log*_2_(1 + *FPKM*)-scale in control PC-3 cell line; PC-3_Mean_l2FC –*log*_2_(*FC*) (fold change) resulting from the shRNA transduction; Correlation_canonical –Spearman’s correlation computed by prostate adenocarcinoma primary tumor samples; Padj –Benjamini-Hochberg adjusted p-value of the zero-correlation test; Seed_canonical –presence of the canonical 6mer seed region; Seed_iso_4 –presence of the +4 5′-isomiR 7mer-m8 seed region; Seed_iso_3 –presence of the +3 5′-isomiR 7mer-m8 seed region.

10.7717/peerj.20642/supp-7 Supplemental Information 7 Jaccard indices for canonical versus +4 5′-isomiR target sets for each analyzed miRNA, including 6mer and 7mer-m8 target counts, intersection and union sizes, and the resulting Jaccard index values 6mer –number of significantly expressed mRNAs containing 6mer seed region of the canonical form; 7mer-m8 –number of significantly expressed mRNAs containing 7mer-m8 seed region of the +4 5′-isomiR; intersection –intersection size of “6mer” and “7mer-m8” mRNAs; union –union size of “6mer” and “7mer-m8” mRNAs; jaccard –intersection / union.

10.7717/peerj.20642/supp-8 Supplemental Information 8 Jaccard indexes for canonical miRNAs and corresponding +3 5′-isoimRs 6mer –number of significantly expressed mRNAs containing 6mer seed region of the canonical form; 7mer-m8 –number of significantly expressed mRNAs containing 7mer-m8 seed region of the +3 5′-isomiR; intersection –intersection size of ”6mer” and ”7mer-m8” mRNAs; union –union size of ”6mer” and ”7mer-m8” mRNAs; jaccard –intersection / union.

10.7717/peerj.20642/supp-9 Supplemental Information 9 Proportion of canonical 6mer seed sites that can be extended to 11-nt matches corresponding to the 7mer-m8 seed of the hsa-miR-93-5p—+4 isomiR, listing 6mer and extended 11mer site counts and their ratios for each miRNA 6mer –number of significantly expressed mRNAs containing 6mer seed region of the canonical form; 7mer-m8 –number of significantly expressed mRNAs extending 6mer seed region with 7mer-m8 seed region of the +4 5′-isomiR; intersection –intersection size of “6mer” and “7mer-m8” mRNAs; union –union size of “6mer” and “7mer-m8” mRNAs; jaccard –intersection / union.

10.7717/peerj.20642/supp-10 Supplemental Information 10 Primer sequences used for miRNA qPCR* * Sequence of canonical has-miR-93-5p (hsa-miR-95-5p|0|0) (5′ → 3′) : CAAAGUGCUGUUCGUGCAGGUAG .

10.7717/peerj.20642/supp-11 Supplemental Information 11 DNA oligonucleotide primers used for site-directed mutagenesis

10.7717/peerj.20642/supp-12 Supplemental Information 12 Raw and processed RT-qPCR data for miR-93 isoform and control miRNA expression in HEK293T control and shMiR-93 cell lines, including Ct values, efficiency calculations, and normalized fold changes used for the analyses in the main text

10.7717/peerj.20642/supp-13 Supplemental Information 13 Raw Ct values and GeNorm-based stability metrics for candidate reference miRNAs (miR-30e-5p, miR-182-5p, miR-22-3p, miR-191-5p, miR-21-5p) across experimental conditions includes efficiency-transformed expression values and average standard deviations used to select normalizers in the main study.

10.7717/peerj.20642/supp-14 Supplemental Information 14 RT-qPCR primer efficiency assessment for the miR-93-5p assay, including dilution-series Ct values, mean Ct per dilution point, and the calculated amplification efficiency (E) with its associated error estimate

10.7717/peerj.20642/supp-15 Supplemental Information 15 RT-qPCR primer efficiency assessment for the miR-182-5p assay, including dilution-series Ct values, mean Ct per dilution point, and the calculated amplification efficiency (E) with its associated error estimate

10.7717/peerj.20642/supp-16 Supplemental Information 16 Melting curve analysis of the RT-qPCR product amplified with miR-191-5p primers in HEK293T control and shMiR-93 cells, indicating a single specific product with uniform Tm across samples and empty controls

10.7717/peerj.20642/supp-17 Supplemental Information 17 Melting curve analysis of the RT-qPCR product amplified with miR-93-5p primers in HEK293T control and shMiR-93 cells, showing a single specific peak and corresponding melting temperatures (Tm)

10.7717/peerj.20642/supp-18 Supplemental Information 18 Melting curve analysis of the RT-qPCR product for the miR-93 UU isoform in HEK293T control and shMiR-93 cells, demonstrating a single specific peak and characteristic Tm values

10.7717/peerj.20642/supp-19 Supplemental Information 19 Melting curve analysis of the RT-qPCR product amplified with miR-182-5p primers in HEK293T control, shMiR-93, and empty vector samples, indicating a single specific product with consistent Tm values

10.7717/peerj.20642/supp-20 Supplemental Information 20 Melting curve analysis of the RT-qPCR product for the miR-93 UUU isoform in HEK293T control and shMiR-93 cells, demonstrating a single specific peak and characteristic Tm values

10.7717/peerj.20642/supp-21 Supplemental Information 21 Translation codebook

## Additional Information and Declarations

### Data Availability

The following information was supplied regarding data availability: The data and code are available in the Supplemental Files.
