# Supplementary figures and images for: Targetome profile of hsa-miR-93-5p is resistant to isoform formation in prostate adenocarcinoma
Source: PeerJ. 2026 Feb 16;14:e20642. doi: 10.7717/peerj.20642 (PMC12919312; doi:10.7717/peerj.20642)

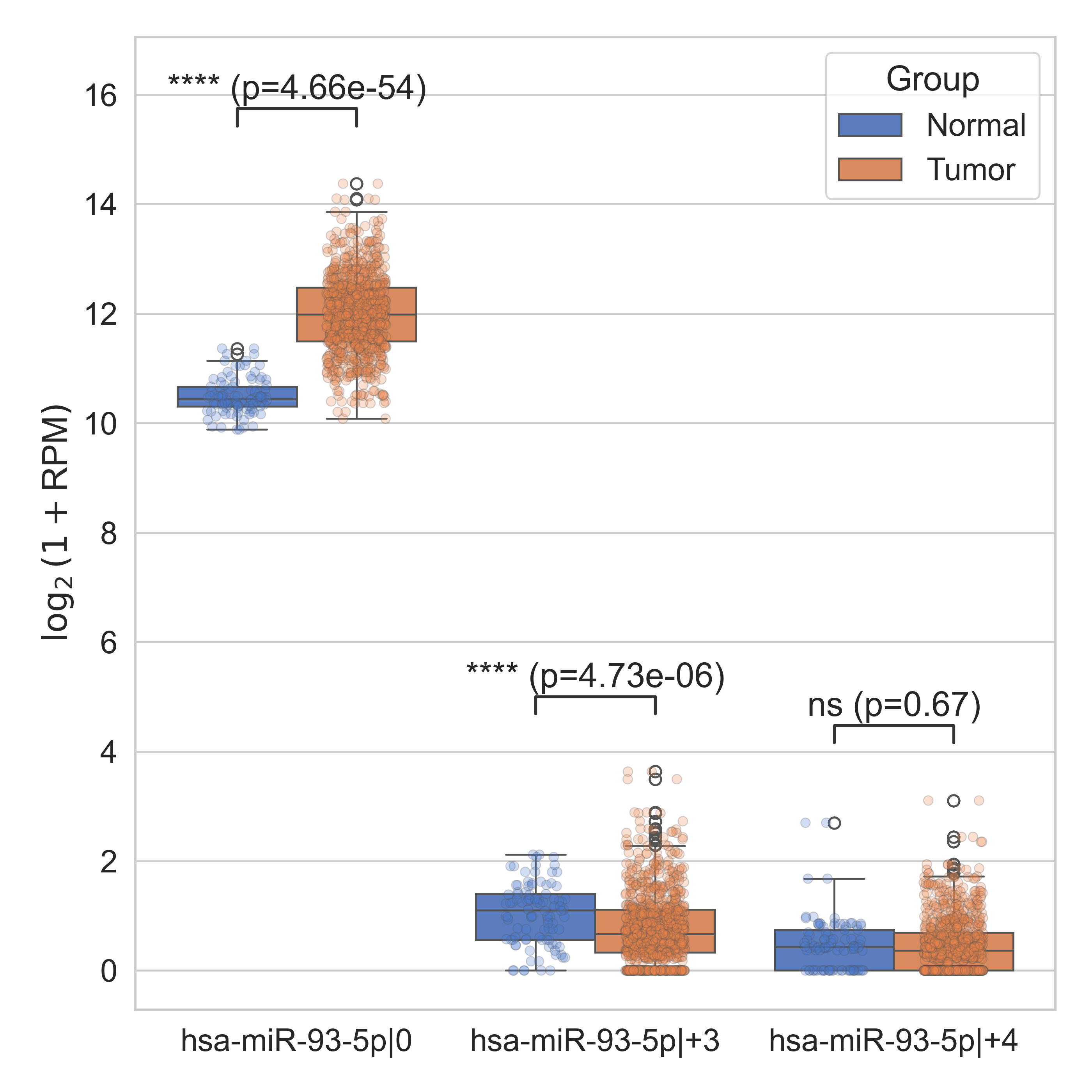

Supplement: Supplemental Information 2 — Differential expression analysis was performed using DESeq2. [file peerj-14-20642-s002.png]

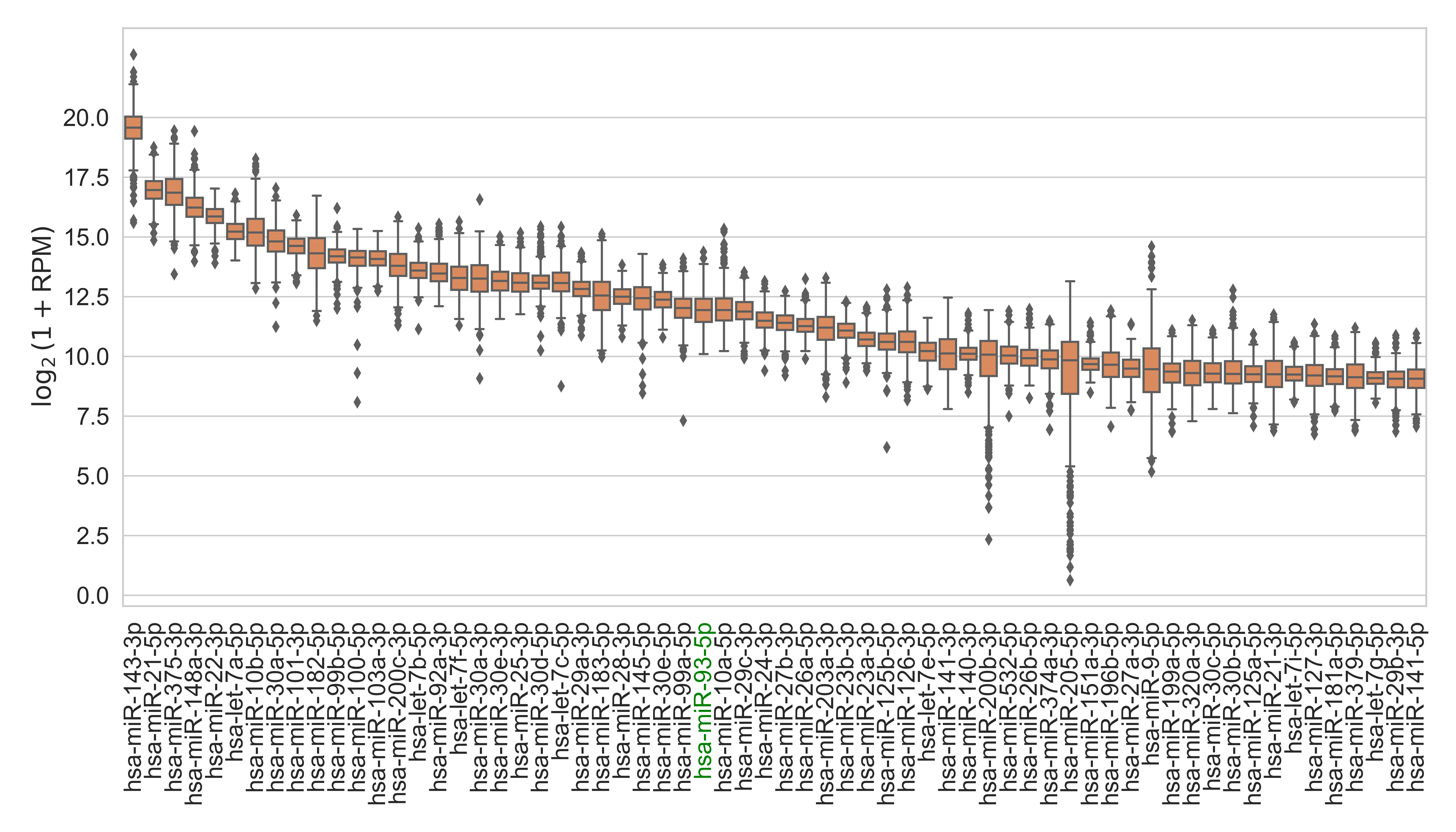

Supplement: Supplemental Information 3 [file peerj-14-20642-s003.png]

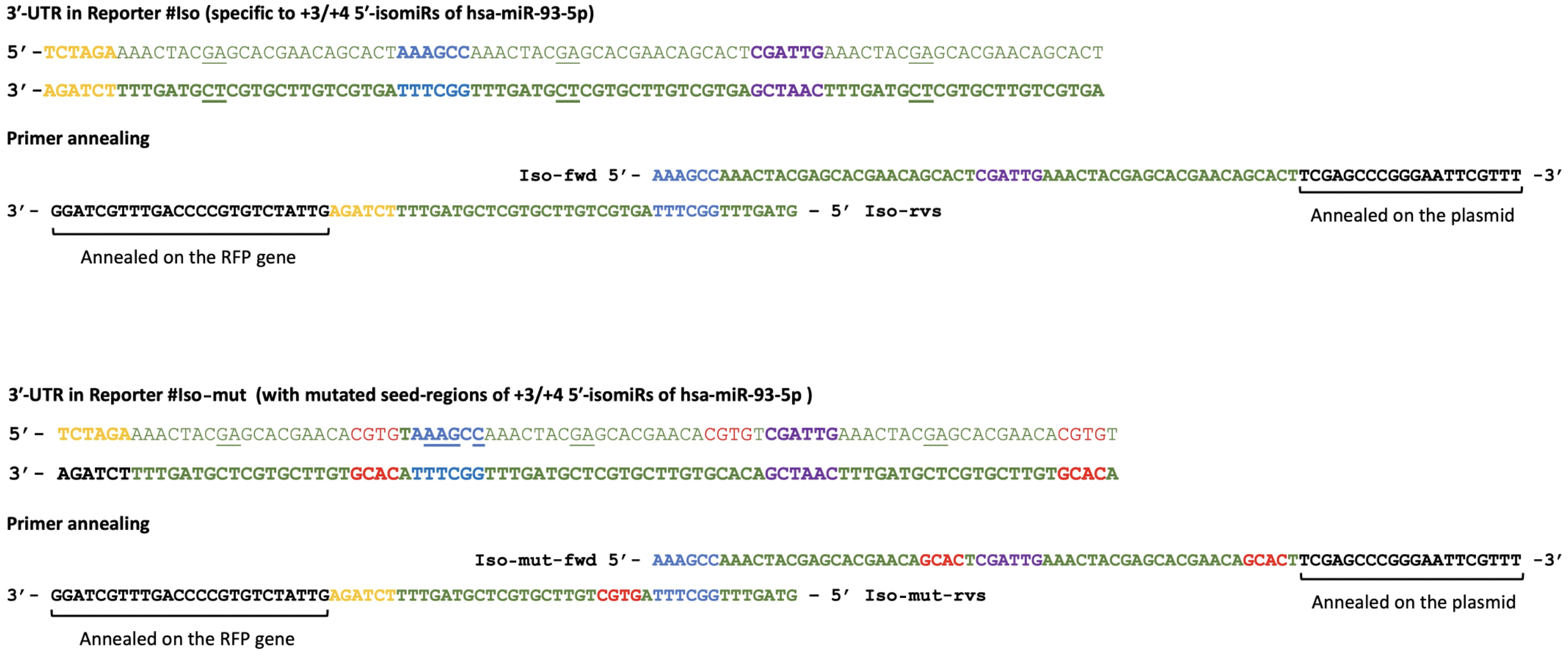

Supplement: Supplemental Information 4 — The 3′-UTR sequences of the reporter constructs used in this study are shown. Also depicted are the annealing patterns of the primers used for site-directed mutagenesis to generate these constructs. Regions that are identical to the +3/+4 5′-isomiRs of hsa-miR-93-5p are highlighted in green and bold. Nucleotides that were intentionally altered to complementary bases –aimed at preventing binding by other miRNAs endogenously and constitutively expressed in HEK293T cells –are shown as underlined text. Spacer sequences separating the binding sites of the +3/+4 5′-isomiRs are highlighted in yellow, blue, and purple. Primer regions complementary to the p.UTA.3.0 plasmid backbone used to construct the reporters are highlighted in black. Mutations introduced into the seed regions to disrupt isomiR binding to the 3′-UTR of the RFP mRNA are highlighted in red. [file peerj-14-20642-s004.png]

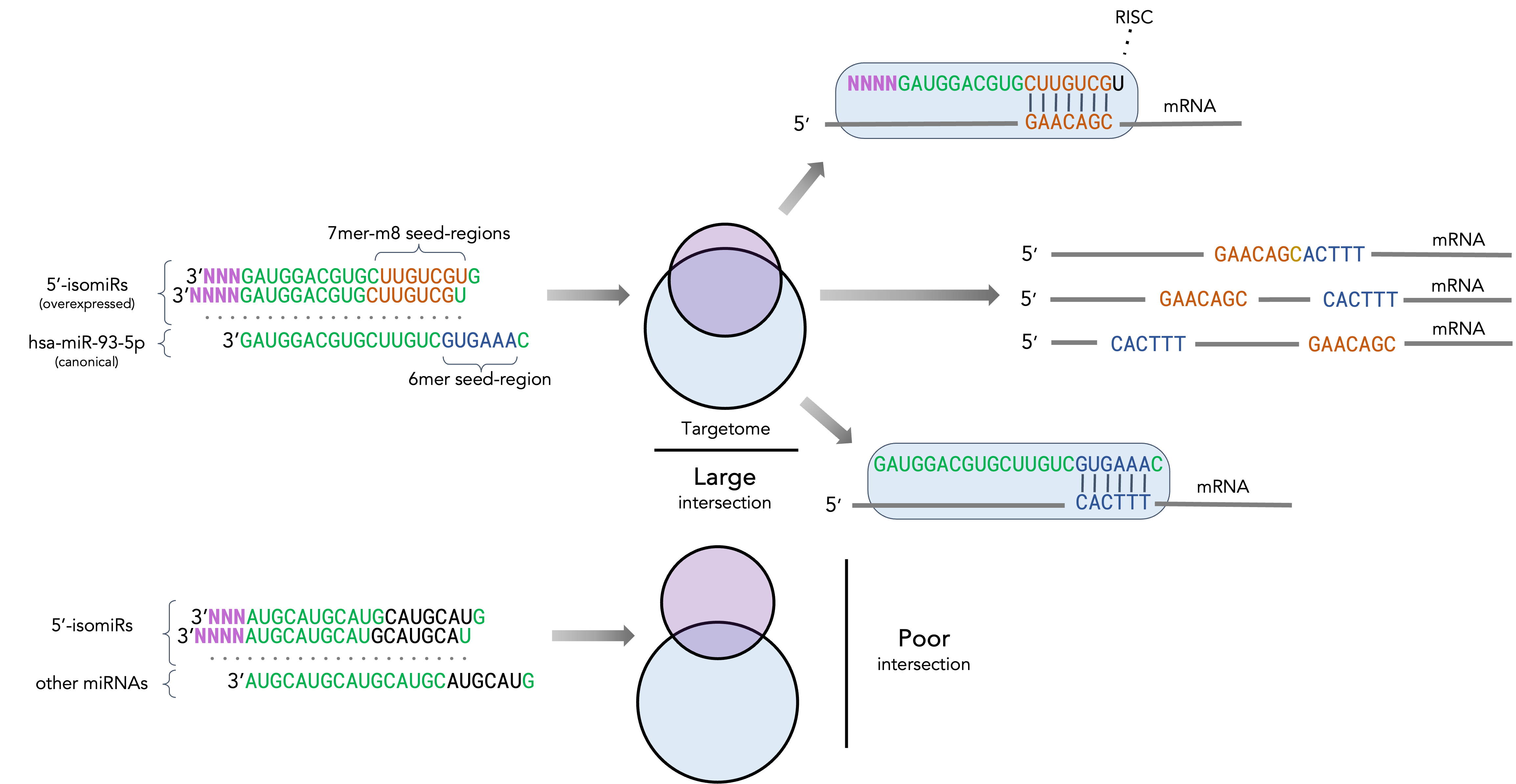

Supplement: Supplemental Information 5 [file peerj-14-20642-s005.png]
