# Supplementary material for: Targetome profile of hsa-miR-93-5p is resistant to isoform formation in prostate adenocarcinoma
Source: PeerJ. 2026 Feb 16;14:e20642. doi: 10.7717/peerj.20642 (PMC12919312; doi:10.7717/peerj.20642)
Supplement: Supplemental Information 10 — * Sequence of canonical has-miR-93-5p (hsa-miR-95-5p|0|0) (5′ → 3′) : CAAAGUGCUGUUCGUGCAGGUAG . [file peerj-14-20642-s010.docx]

**Supplementary Table 5. Primer sequences used for miRNA qPCR***

| **miRNA** | **Forward primer (5'→3')** | **Reverse primer(5'→3')** | **qPCR Efficiency** |
| --- | --- | --- | --- |
| hsa-miR-93-5p  (MIMAT0000093) | GCAAAGTGCTGTTCGTG | CCAGTTTTTTTTTTTTTTTCTACCTG | 1.87 |
| hsa-miR-93-5p\|0\|+2U | GCAAAGTGCTGTTCGTG | GTCCAGTTTTTTTTTTTTTTTAACTACCT |  |
| hsa-miR-93-5p\|0\|+3U | GCAAAGTGCTGTTCGTG | GGTCCAGTTTTTTTTTTTTTTTAAACTCA |  |
| hsa-miR-182-5p | GTTTGGCAATGGTAGAACTCA | GGTCCAGTTTTTTTTTTTTTTTAGTGT | 1.98 |
| hsa-miR-191-5p  (MIMAT0000440) | CAACGGAATCCCAAAAGCA | TCCAGTTTTTTTTTTTTTTTTTTCAGCT | 2.12 |

* Sequence of canonical has-miR-93-5p (hsa-miR-95-5p|0|0) (5'→3'): CAAAGUGCUGUUCGUGCAGGUAG
