# Supplementary material for: Targetome profile of hsa-miR-93-5p is resistant to isoform formation in prostate adenocarcinoma
Source: PeerJ. 2026 Feb 16;14:e20642. doi: 10.7717/peerj.20642 (PMC12919312; doi:10.7717/peerj.20642)
Supplement: Supplemental Information 11 [file peerj-14-20642-s011.docx]

**Supplementary Table 6. DNA oligonucleotide primers used for site-directed mutagenesis**

| **Primer designation** | **Primer sequence (5'→3')** |
| --- | --- |
| *Used for the reporter specific to hsa-miR-93-5p\|+3/+4 5′-isomiRs (#Iso)* | |
| Iso-fwd | AAAGCCAAACTACGAGCACGAACAGCACTCGATTGAAACTACGAGCACGAACAGCACTTCGAGCCCGGGAATTCGTTT |
| Iso-rvs | GTAGTTTGGCTTTAGTGCTGTTCGTGCTCGTAGTTTTCTAGAGTTATCTGTGCCCCAGTTTGCTAGG |
| *Used for the reporter with mutated seed-regions of hsa-miR-93-5p\|+3/+4 5′-isomiRs (#Iso-mut)* | |
| Iso-mut-fwd | AAAGCCAAACTACGAGCACGAACACGTGTCGATTGAAACTACGAGCACGAACACGTGTTCGAGCCCGGGAATTCGTTT |
| Iso-mut-rvs | TAGTTTGGCTTTACACGTGTTCGTGCTCGTAGTTTTCTAGAGTTATCTGTGCCCCAGTTTGCTAGG |
