# Supplementary material for: Targetome profile of hsa-miR-93-5p is resistant to isoform formation in prostate adenocarcinoma
Source: PeerJ. 2026 Feb 16;14:e20642. doi: 10.7717/peerj.20642 (PMC12919312; doi:10.7717/peerj.20642)
Supplement: Supplemental Information 16 [file peerj-14-20642-s016.pdf]

Отчёт по результатам анализа ПЦР

11-Jul-25

ДНК-ТЕХНОЛОГИЯ

Настройки анализа: метод: Пороговый (Ct) (BF), cr=9, vt=10, tp=30, tv=5

Дата: 21.05.2024, 13:59:31
Номер протокола: 0
Оператор: Гость
Файл с результатами: Roma 21.05.r96
Комментарий:

Тест: **TEST**

Программа амплификации: **60__evrogen (25мкл)**

1. 95.0 °C - 0:05:00
2. 94.0 °C - 0:00:20] *40
   60.0 °C - 0:00:10
   72.0 °C - 0:00:15
3. 56.0 °C - 0:00:06 Δt] *200 (0.20)
4. 10.0 °C - хранение

Кривая плавления

Номер лунки	Идентификатор пробирки	Tm Fam	Tm Hex
F1	HEK293T-shMiR-93_miR-191 (TES	73.0	
F2	HEK293T-shMiR-93_miR-191 (TES	73.0	
F3	HEK293T-shMiR-93_miR-191 (TES	73.1	
F4	HEK293T-Ctrl_miR-191 (TEST)	73.0	
F5	HEK293T-Ctrl_miR-191 (TEST)	73.0	
F6	HEK293T-Ctrl_miR-191 (TEST)	73.1	
F10	Empty_miR-191 (TEST)	73.5	
F11	Empty_miR-191 (TEST)	73.6	
F12	Empty_miR-191 (TEST)	73.6	

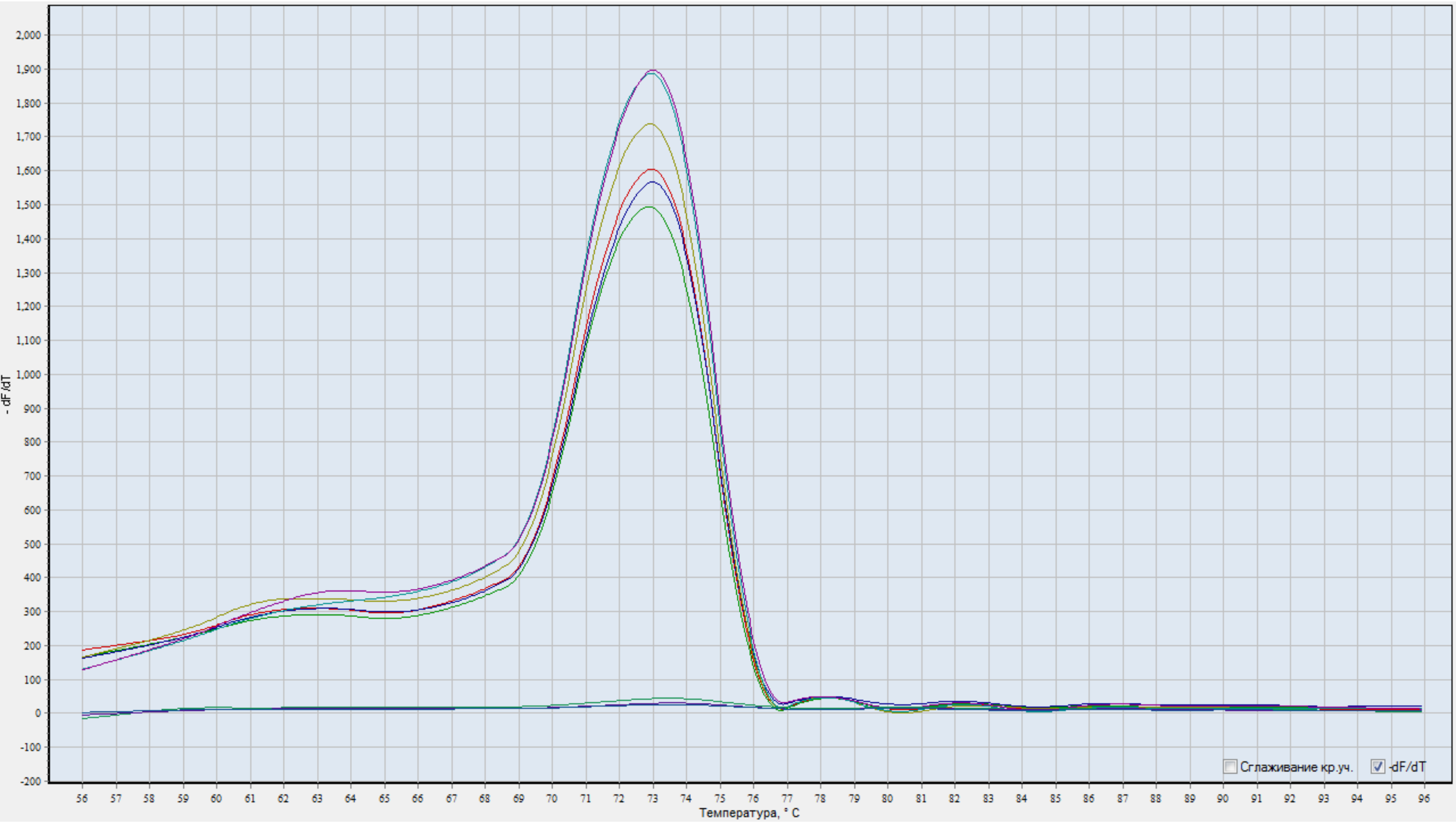

- dF/dT
2,000
1,900
1,800
1,700
1,600
1,500
1,400
1,300
1,200
1,100
1,000
900
800
700
600
500
400
300
200
100
0
-100
-200
56 57 58 59 60 61 62 63 64 65 66 67 68 69 70 71 72 73 74 75 76 77 78 79 80 81 82 83 84 85 86 87 88 89 90 91 92 93 94 95 96
Температура, ° C
Сглаживание кр.уч.
-dF/dT
