# Supplementary material for: Targetome profile of hsa-miR-93-5p is resistant to isoform formation in prostate adenocarcinoma
Source: PeerJ. 2026 Feb 16;14:e20642. doi: 10.7717/peerj.20642 (PMC12919312; doi:10.7717/peerj.20642)
Supplement: Supplemental Information 18 [file peerj-14-20642-s018.pdf]

Отчёт по результатам анализа ПЦР

11-Jul-25

ДНК-ТЕХНОЛОГИЯ

Настройки анализа: метод: Пороговый (Ct) (BF), cr=9, vt=10, tp=30, tv=5

Дата: 21.05.2024, 13:59:31
Номер протокола: 0
Оператор: Гость
Файл с результатами: Roma 21.05.r96
Комментарий:

Тест: **TEST**

Программа амплификации: **60__evrogen (25мкл)**

1. 95.0 °C - 0:05:00
2. 94.0 °C - 0:00:20] *40
   60.0 °C - 0:00:10
   72.0 °C - 0:00:15 📷
3. 56.0 °C - 0:00:06 📷Δt] *200 (0.20)
4. 10.0 °C - хранение

Кривая плавления

Номер лунки	Идентификатор пробирки	Tm Fam	Tm Hex
C1	HEK293T-shMiR-93_miR-93_UU (T	74.0	
C2	HEK293T-shMiR-93_miR-93_UU (T	74.0	
C3	HEK293T-shMiR-93_miR-93_UU (T	74.0	
C4	HEK293T-Ctrl_miR-93_UU (TEST)	74.0	
C5	HEK293T-Ctrl_miR-93_UU (TEST)	74.1	
C6	HEK293T-Ctrl_miR-93_UU (TEST)	74.1	
C10	Empty_miR-93_UU (TEST)	73.8	
C11	Empty_miR-93_UU (TEST)	73.7	
C12	Empty_miR-93_UU (TEST)	73.6	

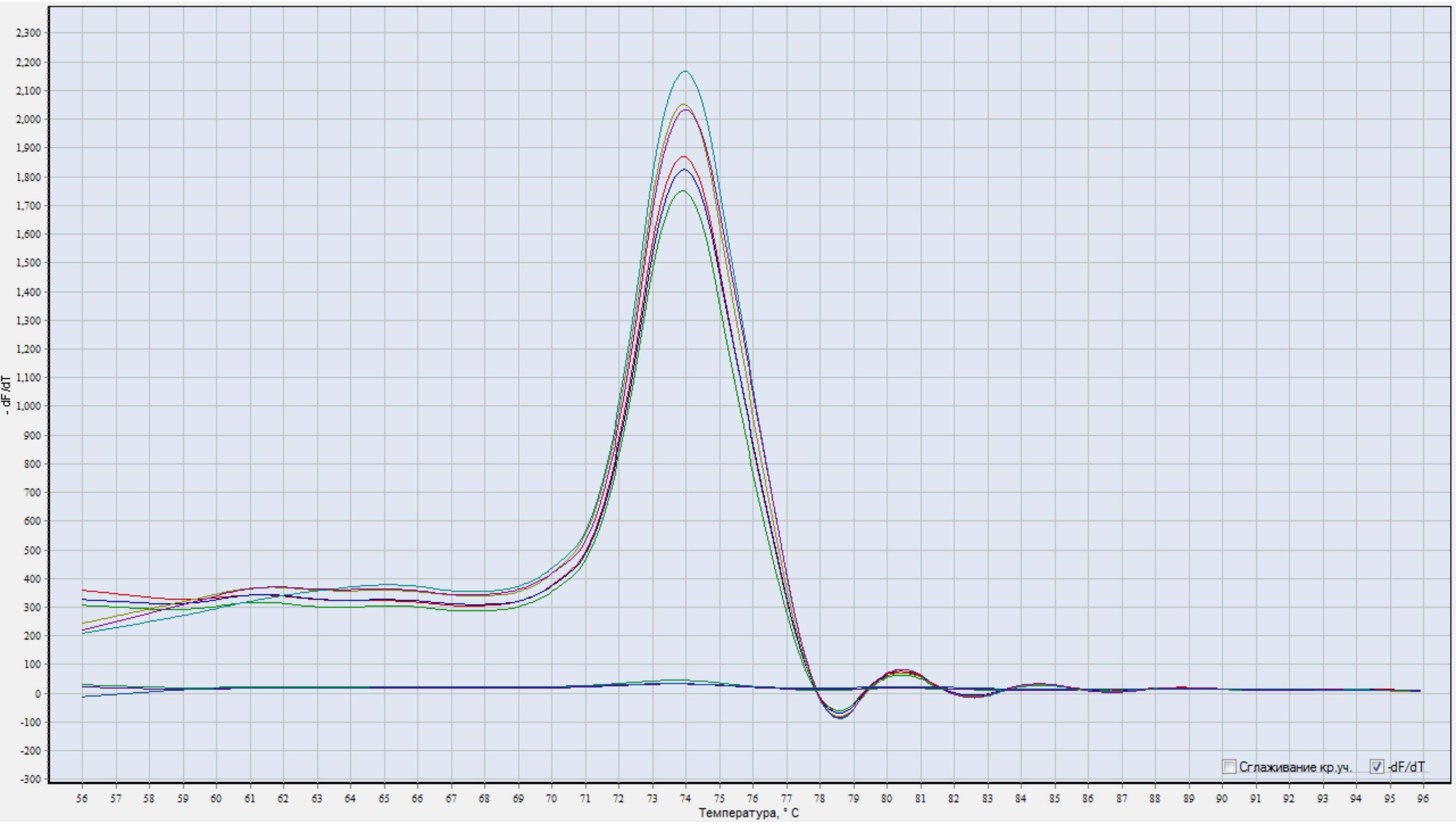

- dF/dT
Температура, ° C
Сглаживание кр.уч.
-dF/dT
