# Supplementary material for: Targetome profile of hsa-miR-93-5p is resistant to isoform formation in prostate adenocarcinoma
Source: PeerJ. 2026 Feb 16;14:e20642. doi: 10.7717/peerj.20642 (PMC12919312; doi:10.7717/peerj.20642)
Supplement: Supplemental Information 20 [file peerj-14-20642-s020.pdf]

Отчёт по результатам анализа ПЦР

11-Jul-25

ДНК-ТЕХНОЛОГИЯ

Настройки анализа: метод: Пороговый (Ct) (BF), cr=9, vt=10, tp=30, tv=5

Дата: 21.05.2024, 13:59:31
Номер протокола: 0
Оператор: Гость
Файл с результатами: Roma 21.05.r96
Комментарий:

Тест: **TEST**

Программа амплификации: **60__evrogen (25мкл)**

1. 95.0 °C - 0:05:00
2. 94.0 °C - 0:00:20] *40
   60.0 °C - 0:00:10
   72.0 °C - 0:00:15 📷
3. 56.0 °C - 0:00:06 📷Δt] *200 (0.20)
4. 10.0 °C - хранение

Кривая плавления

Номер лунки	Идентификатор пробирки	Tm Fam	Tm Hex
D1	HEK293T-shMiR-93_miR-93_UUU	74.4	
D2	HEK293T-shMiR-93_miR-93_UUU	74.4	
D3	HEK293T-shMiR-93_miR-93_UUU	74.4	
D4	HEK293T-Ctrl_miR-93_UUU (TES	74.3	
D5	HEK293T-Ctrl_miR-93_UUU (TES	74.3	
D6	HEK293T-Ctrl_miR-93_UUU (TES	74.3	
D10	Empty_miR-93_UUU (TEST)	73.7	
D11	Empty_miR-93_UUU (TEST)		
D12	Empty_miR-93_UUU (TEST)		

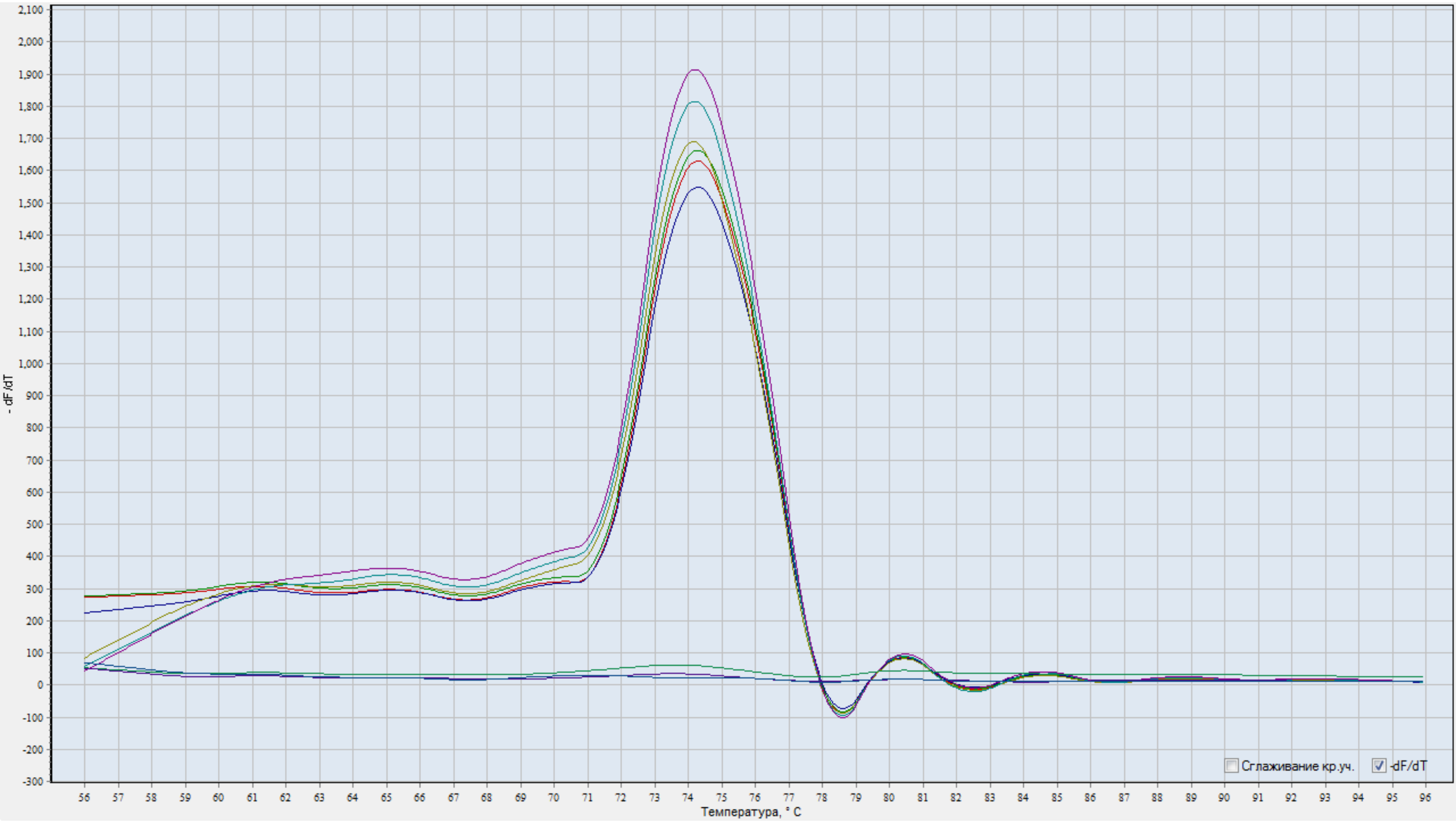

2,100
2,000
1,900
1,800
1,700
1,600
1,500
1,400
1,300
1,200
1,100
1,000
900
800
700
600
500
400
300
200
100
0
-100
-200
-300
- dF/dT
56 57 58 59 60 61 62 63 64 65 66 67 68 69 70 71 72 73 74 75 76 77 78 79 80 81 82 83 84 85 86 87 88 89 90 91 92 93 94 95 96
Температура, ° C
Сглаживание кр.уч.
-dF/dT
